# Computational Identification of Metabolic Pathways of *Plasmodium falciparum* using the *k*-Shortest Path Algorithm

**DOI:** 10.1155/2019/1750291

**Published:** 2019-10-01

**Authors:** Jelili Oyelade, Itunuoluwa Isewon, Olufemi Aromolaran, Efosa Uwoghiren, Titilope Dokunmu, Solomon Rotimi, Oluwadurotimi Aworunse, Olawole Obembe, Ezekiel Adebiyi

**Affiliations:** ^1^Department of Computer & Information Sciences, Covenant University, Ota, Nigeria; ^2^Covenant University Bioinformatics Research Cluster (CUBRe), Ota, Nigeria; ^3^Department of Biochemistry, Covenant University, Ota, Nigeria; ^4^Department of Biological Sciences, Covenant University, Ota, Nigeria

## Abstract

*Plasmodium falciparum*, a malaria pathogen, has shown substantial resistance to treatment coupled with poor response to some vaccines thereby requiring urgent, holistic, and broad approach to prevent this endemic disease. Understanding the biology of the malaria parasite has been identified as a vital approach to overcome the threat of malaria. This study is aimed at identifying essential proteins unique to malaria parasites using a reconstructed *iPfa* genome-scale metabolic model (GEM) of the 3D7 strain of *Plasmodium falciparum* by filling gaps in the model with nineteen (19) metabolites and twenty-three (23) reactions obtained from the MetaCyc database. Twenty (20) currency metabolites were removed from the network because they have been identified to produce shortcuts that are biologically infeasible. The resulting modified *iPfa* GEM was a model using the *k*-shortest path algorithm to identify possible alternative metabolic pathways in glycolysis and pentose phosphate pathways of *Plasmodium falciparum*. Heuristic function was introduced for the optimal performance of the algorithm. To validate the prediction, the essentiality of the reactions in the reconstructed network was evaluated using betweenness centrality measure, which was applied to every reaction within the pathways considered in this study. Thirty-two (32) essential reactions were predicted among which our method validated fourteen (14) enzymes already predicted in the literature. The enzymatic proteins that catalyze these essential reactions were checked for homology with the host genome, and two (2) showed insignificant similarity, making them possible drug targets. In conclusion, the application of the intelligent search technique to the metabolic network of *P. falciparum* predicts potential biologically relevant alternative pathways using graph theory-based approach.

## 1. Introduction

**Figure 1:**
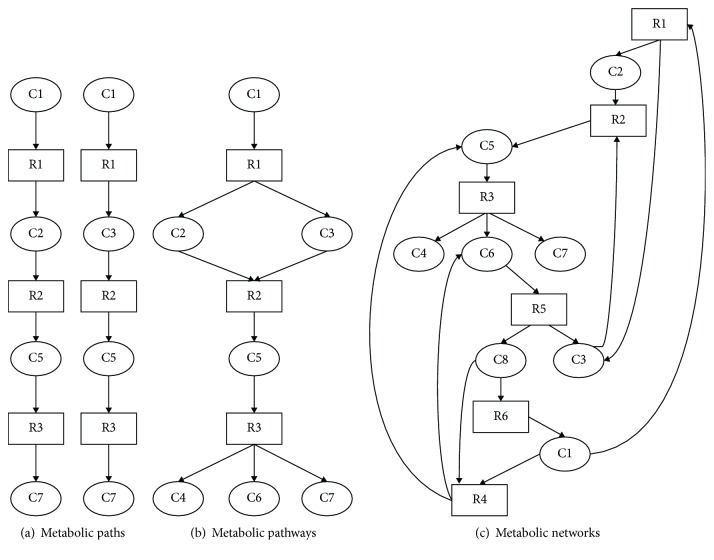
(a) describes the paths in a metabolic network construction; (b) describes the pathways; and (c) describes the entire networks of the metabolic construction.

Malaria remains one of the leading global health challenges with about 216 million cases and more than 445,000 deaths recorded in 2016. According to the World Health Organization (WHO), 88% of these deaths occurred in Africa [1] and *Plasmodium falciparum* accounted for the majority of the cases. *P. falciparum* has developed resistance to all antimalarial medications including the most potent one—artemisinin [2–4]. Some genetic changes and metabolic alterations confer survival advantage that enables the parasite to evade drug effects thereby ensuring its survival. However, there is still a poor understanding of the processes utilized by *P. falciparum* to evade drug effects, which also hamper vaccine development [5]. The incomplete knowledge of the metabolic pathways of *P. falciparum* has also been identified as a major impediment towards the development of an effective treatment [6]; hence, the need for intensified research that is aimed at understanding better the parasite biology. Computational approach, which predicts metabolic networks and possible alternative pathways pertinent to the parasite's survival, can identify candidate drug targets to eliminate the disease globally. Some previous studies have been done in this regard.  Figure 1 explains the various paths (a), pathways (b), and complete network constructions (c) in metabolic pathways.

The identification of these undiscovered pathways within the metabolism of the malaria parasite entails enumerating not only the shortest path within the metabolic pathway but also the list of other feasible paths within a source compound and a target compound. This could be regarded as an optimization problem. The metabolic system or metabolism of a specific cell or an organism is the entire system of metabolic reactions of the cell or organism. A metabolic pathway is an associated subsystem of the metabolic system either as a representation of particular processes or characterized by functional boundaries, e.g., the system between a glucose (initial substance) and a pyruvate (final substance). The *k*-shortest path technique enables the optimal and suboptimal metabolic pathways to be identified within the metabolic network [7].

Croes et al. [8] presented a reaction to the representation of the metabolic network in order to identify relevant pathways in a biological network. However, the path finding algorithm applied was unable to handle a large dataset. Faust et al. [9] combined a random walk-based reduction of the graph with the shortest path-based algorithm and applied on a yeast metabolic network. This approach is computationally intensive due to several runs by the shortest path algorithm used. Oyelade et al. [10] applied a colour coding algorithm to search for minimum pathways in the *P. falciparum* interaction network. They discovered “identified” and “unknown” genes and signal transduction pathway involved in metabolic activities of *P. falciparum*. However, this approach is only capable of obtaining the shortest signal pathway. Also, in another work of Oyelade et al. [11], the qualitative Petri net model for the glycolysis pathway in P. *falciparum* was built and analysed for its structural and quantitative properties using the Petri net theory which only give insights into the complex net behavior of the pathway. Essential reactions in the metabolic network of *P. falciparum* have been predicted using several computational techniques such as *in silico* knockout screening [12], load and choke point [13], centrality measures [14], flux balance analysis [15], and machine learning approach [16] etc.

The goal of this study is to computationally identify branching metabolic pathways of *P. falciparum* using the enhanced *k*-shortest path technique with improved prediction precision. This technique was used in our previous work [17] although limited to finding only alternative paths in selected metabolic pathways. In this work, we obtained the genome-scale metabolic model of the 3D7 strain of *P. falciparum* from a previous study [18]. The dataset contains 325 genes and 670 metabolic reactions. The underlying architecture of the metabolic network was modelled using a reaction graph where reactions are designated as the nodes and two reactions are neighbors, if the product of a reaction is the substrate of the other. Compound graph representation is not suitable for path finding algorithms because a reaction can have more than one compound as both input and output, e.g., A + B ≥ C + D, thereby leading to a bunch of redundant edges. The existence of two different types of nodes in a bipartite graph makes it complex to traverse by the path finding algorithm. The choice of a reaction graph was informed by the aim of this study, which is to identify more alternative metabolic pathways, and the pathways are known to be chains of reactions and also due to the applicability of path finding algorithms which requires a single type of node to compute the shortest paths within the network. The *T*∗ algorithm, the *k*-shortest path technique developed by Kadivar [19], was adapted to extract the shortest paths from a defined source node to a target node. Only two metabolic pathways were considered for the alternative path analysis in this work due to the overlapping nature of the pathways that made it difficult to identify source and target reactions relating to a specific pathway.

We set the value of *k* of the path finding algorithm to equal five (5) because as *k* increases, the biological relevance of the path reduces. The identification of essential enzymes in a particular network allows a possible drug target to be identified [16, 20–22]. Therefore, in drug development, essential enzymes are generally recognized as perfect drug target candidates for potential new drugs and vaccines to treat and prevent diseases since their deletion from a network can compromise its integrity [21, 22]. Hence, the essentiality of reactions was carried out using the betweenness centrality measure to determine essential reactions within a reconstructed network and subsequently predicted 32 essential enzymes.

## 2. Materials and Methods

### 2.1. Reconstructing the Metabolic Network

Metabolic reaction data were obtained from Chiappino-Pepe et al.'s study [18]; also 19 metabolites and 23 reactions were obtained from the MetaCyc database [23] to fill gaps in the *iPfa* GEM (see appendix A for the list of reactions obtained from the MetaCyc database). In a metabolic network, there are several metabolites that are commonly involved in reactions that cause shortcuts without biological meaning when computing paths in a simple graph [24]. These metabolites are referred to as pool metabolites or currency metabolites such as proton and water. Most of them often produce biological misinterpretations due to the artifactual links between nodes.

Kim et al. [25] identified twenty-five (25) currency metabolites out of which we eliminated twenty (20) from the network before reconstructing the graph. The remaining five (5) currency metabolites identified by [16] were left in the network since their presence does not have a significant effect that could lead to shortcuts without biological meaning, and a uniform weight is assigned to all edges in the network.

### 2.2. The Algorithm

**Figure 2:**
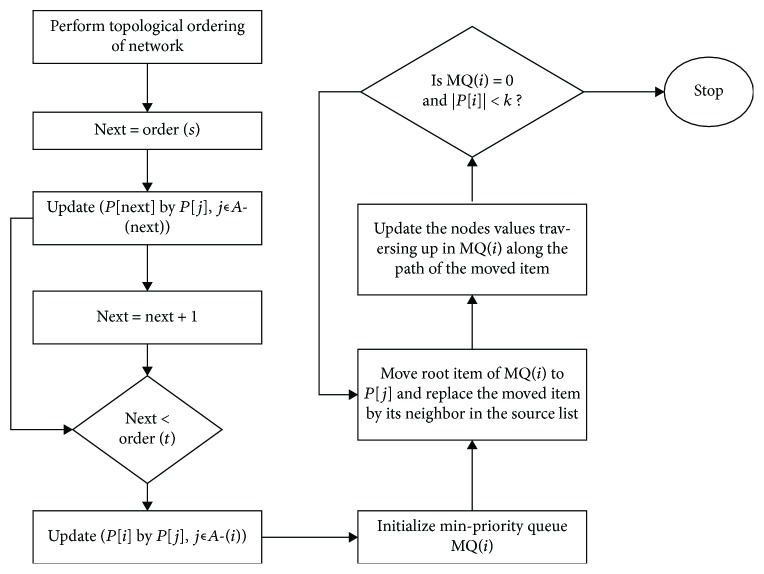
*T*∗ algorithm flow chart for metabolic network reconstruction.

The *T*∗ algorithm was selected among the other *k*-shortest paths because of its superior computational performance (as shown in Table 1). This algorithm requires topological sorting of the graph nodes as its input, which is only possible for a directed acyclic graph.  Figure 2 shows the algorithm flow chart of the metabolic network construction.

Given that *P*[*i*] = {*P*_1_^*i*^, *P*_2_^*i*^, ⋯, *P*_*k*_^*i*^}as the set of the *k*_*i*_-shortest paths from *s* to node *i* and *L*(*P*_1_^*i*^) ≤ *L*(*P*_2_^*i*^) ≤ *L*(*P*_*k*_^*i*^), where *L*(*P*) denotes the length of path *P*. Let (*i*, *j*) ∈ *A*, because of topological ordering and since *order* (*j*) is larger than the other nodes in all *s*–*i* paths; *P*_*l*_^*i*^ ⋃ {(*i*, *j*)} is a loopless path for each *l* ∈ {1, 2, ⋯, *k*_*i*_}. Therefore if *P*[*s*] = ∅, then *P*[*i*] = ⋃_*j*∈*A*−(*i*)_⋃_*P*_*ϵ*_*P*∣*j*∣_ *P* ⋃ {(*j*, *i*)} (stated in Algorithm 1).

**Figure 3:**
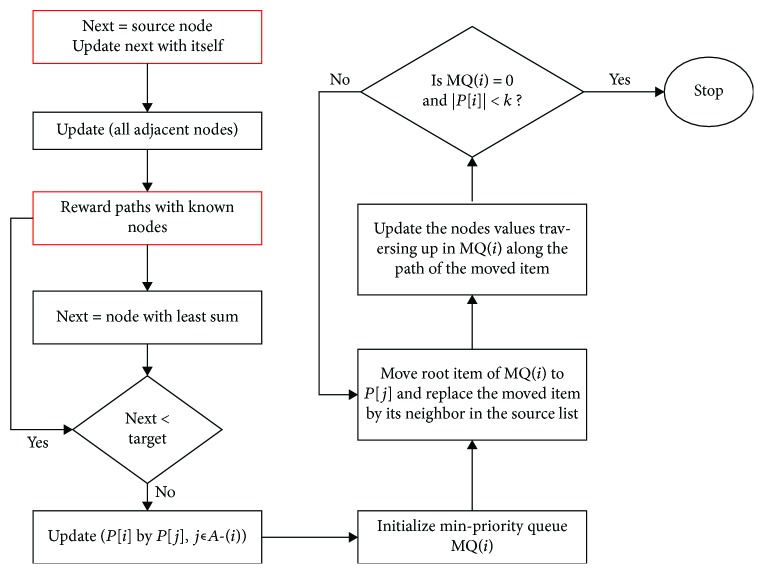
Modified *T*^∗^ algorithm flow chart for metabolic network reconstruction.

The *T*∗ algorithm was modified in two significant ways:

Firstly, we eliminated the first stage (topological ordering) due to the nature of the dataset where several reactions within the dataset could be on the same level in the topological order thereby varying the results from each run of the algorithm. For instance, if reactions A, B, and C have the same topological order, then it implies that at different runs of the algorithm, reaction A could come first or reaction B or reaction C, which varies the result of the algorithm at different runs. The metabolic network of *P. falciparum* contains over six hundred compounds and over a thousand reactions. The inconsistency inherent in the ordering of the reactions will have severe biological implication for the eventual shortest paths generated by the algorithm. For instance, if the order of a vital reaction within the pathway is less than that of the source compound for a particular pathway, then the reaction will be omitted from the shortest paths because the algorithm starts traversing from the source node to the target node in the topological order. Secondly, the topological ordering is not applicable to networks with a cycle since metabolic networks are known to contain loops; hence, we introduced heuristic function to enhance the prediction precision of the alternative paths.  Figure 3 shows the modified *T*^∗^ algorithm for metabolic network construction. The reactions in the annotated pathway were retrieved and used to guide the search of the algorithm to list biological feasible paths.

Therefore, the modified *T*^∗^algorithm 2 is given.

#### 2.2.1. Reward Shaping Formulation

The concept of reward shaping was also introduced to the *k*-shortest path algorithm to improve the quality of biologically relevant alternative path prediction. Reward shaping is the addition of an extra reward signal that encodes some heuristic knowledge of the system designer or domain expert, thus encouraging the learning agent to explore parts of the state space believed to contain good solutions [26–28]. In practice, the choice of reward function is intuitively selected [29] and this has been successfully applied to speed up reinforcement learning techniques in complex domains [30, 31].

The reward *R*_*k*_ of each feasible path between a source reaction and target reaction is computed using the number of reactions within the path that can be found in the set of annotated reactions *R*_*A*_. An annotated pathway was obtained from the KEGG database. The justification for this model is based on the assumption that the annotated pathway for a metabolic process represents the shortest path; hence, the next shortest alternative path is most likely to have branched from the annotated path thereby containing most of the reactions in annotation. The reactions in the feasible path were compared to the annotated path to determine the number of intersecting reactions. However, the total cost of a path is a function of its length and the number of annotated reaction contained in the path.

(1) $$\begin{array}{c} M_{k}=\overset{n}{\underset{i=1}{\sum }}x_{i}c\left(r_{i}\right),\;x=\left\{\begin{array}{c} 1,\;\text{if }r_{i}\;\epsilon \;R_{A},\\ 0,\;\text{otherwise}, \end{array}\right. \end{array}$$

where *M*_*k*_ represents the initial reward for path *k*, *c*(*r*_*i*_) represents the coefficient for reaction (*r*_*i*_) which has a constant value of 1. *x*_*i*_ will assume the value of 1 if *r*_*i*_ is present in the set of annotated reaction for that pathway and 0 if otherwise.

In addition to the objective, obtaining biologically relevant paths is also to obtain the shortest path; hence, a penalty function is introduced which is meant to reduce the reward of paths based on their length; this is represented in

(2) $$\begin{array}{c} Pk=\frac{n_{k}}{t}, \end{array}$$

where *t* is a “balancing factor” which is an arbitrary value that is suitable to provide a penalty value that balances the positiveness of annotated reactions present in the path and the disadvantages of the path length.

The reward for a path *k* is given as the difference between its initial reward and penalty score as shown in

(3) $$\begin{array}{c} Rk=Mk-Pk. \end{array}$$

In this study, we chose *t* to be 0.5 for a fair penalty score and the adjusted cost is the difference between the number of reaction in the path and the reward. We then applied the modified *T*^∗^ algorithm to the glycolysis and pentose phosphate pathway of *P. falciparum* to obtain the *k*-shortest paths for *k* = 5.

## 3. Results

**Figure 4:**
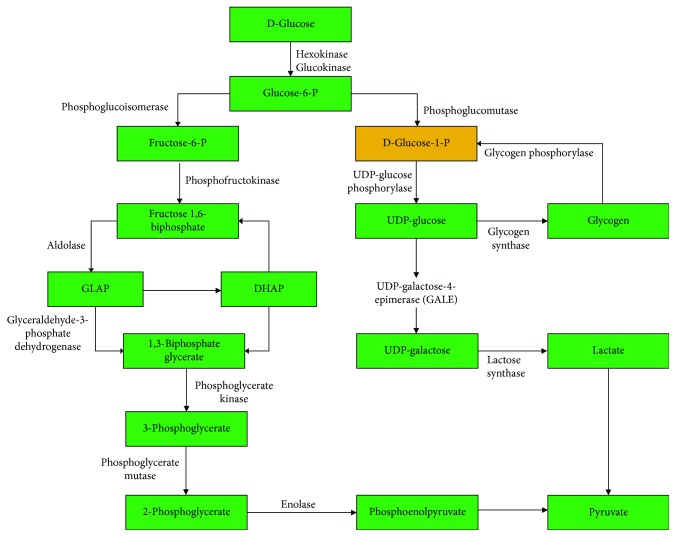
Predicted alternative metabolic paths in the glycolysis pathway. The compound (D-glucose-1-P) highlighted in gold represents the predicted alternative path to the final product (pyruvate).

**Figure 5:**
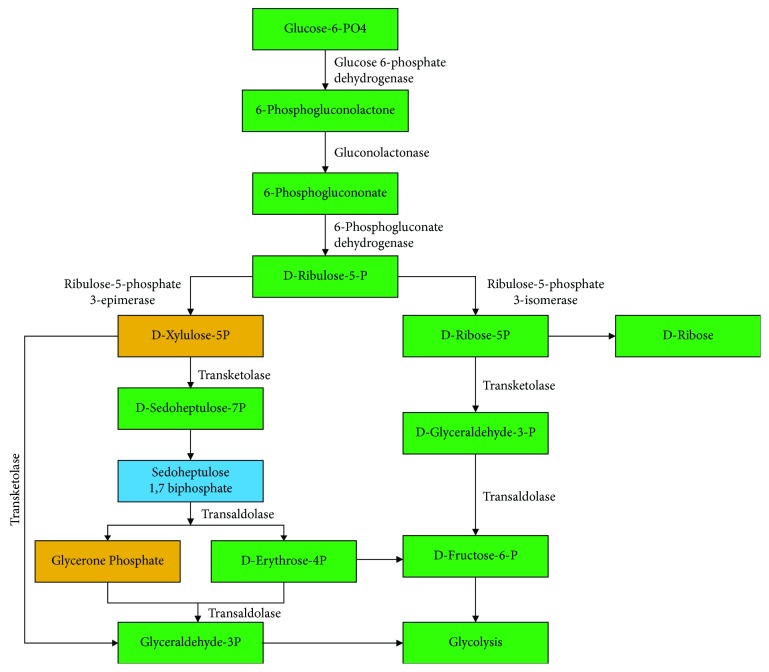
Predicted alternative metabolic paths in the pentose phosphate pathway. The compounds (D-xylulose-5P and glycerone phosphate) highlighted in gold represent the predicted alternative paths to the final product. The compound (sedoheptulose 1,7 biphosphate) highlighted in blue is the predicted nonannotated compound in the pathway.

We then applied the modified *T*^∗^ algorithm to the glycolysis and pentose phosphate pathway of *P. falciparum* to obtain the *k*-shortest paths for *k* = 5.  Table 2 shows the number of reactions present in a predicted path and its computed cost. The five alternative paths identified by our method for each of the target metabolic pathways were further verified manually to identify artifacts and biologically plausible paths. Two alternative paths were identified to be a feasible path for metabolic activities in the pentose phosphate pathway. One alternative path was identified in the glycolysis pathway, which could serve as an alternative path for metabolic activities in the pathway. The reactions represented in gold in Figures 4 and 5 indicate reactions identified by this study to have created alternative paths in the respective metabolic pathways. The reaction represented in blue in Figure 5 represents a nonannotated reaction identified by this study to create an alternative path glucose 6-P to glycolysis.

For the glycolysis pathway, we predicted a pathway, which could provide an alternative path for the generation of pyruvate. This metabolic reaction involves alpha-D-glucose-1-phosphate uridylyltransferase. The predicted alternative paths for the glycolysis pathway are presented in Figure 4.

The predicted alternative paths for the pentose phosphate pathway are presented in Figure 5. Two (2) additional metabolic reactions were predicted to have created an alternative path in the pathway; they are D-glyceraldehyde-3-phosphate glycolaldehyde transferase and sedoheptulose 7-phosphate 1-phosphotransferase reactions, respectively.

### 3.1. Essential Reaction Prediction

To verify the predicted pathways, a test of the essentiality of these genes was done on the predicted pathways. Betweenness centrality measure was applied to the reconstructed metabolic network to each reaction in the network to determine the essentiality of a particular metabolic pathway that is critical for the production of a target compound identified. The betweenness centrality method is stated as follows:

(4) $$\begin{array}{c} b_{ik}\left(p_{j}\right)=\frac{g_{ik}\left(p_{j}\right)}{g_{ik}}, \end{array}$$

where *b*_*ik*_(*p*_*j*_) is the proportion of the shortest path linking *p*_*i*_ to *p*_*k*_ that contain *p*_*j*_, *g*_*ik*_(*p*_*j*_) is the number of the shortest path that contain point *p*_*j*_ as an intermediary in the shortest path from *p*_*i*_ to *p*_*k*_, and *g*_*ik*_ is the number of the shortest path from *p*_*i*_ to *p*_*k*_.

(5) $$\begin{array}{c} {d_{ij}}^{*}=\overset{n}{\underset{k=1}{\sum }}b_{ik}\left(p_{j}\right)\;\left(i\neq j\neq k\right), \end{array}$$

where *d*_*ij*_ is the pair dependency which represents the degree to which a point, *p*_*i*_, must depend upon another, *p*_*j*_.

(6) $$\begin{array}{c} C_{B}\left(p_{j}\right)=\overset{n}{\underset{i=1}{\sum }}{\overset{*}{d}}_{ij}, \end{array}$$

where *C*_*B*_(*p*_*j*_) is the betweenness point centrality.

The genes that coded the enzymes responsible for the essential reactions discovered were obtained and blasted against the human genome to evaluate the existence of homology in the host. We used a centrality score of 0.25 as the level of significance because the centrality score of some of our predicted reactions that validated the gold standard set of essential enzymes and other predicted ones in literature has a centrality score of approximately 0.3.  Table 3 shows the reactions with a centrality score from 0.25 and above as the predicted essential reactions. A total of 32 reactions were predicted as essential, out of which 13 have been validated in the literature.

### 3.2. Visualization of Predicted Pathway

**Figure 6:**
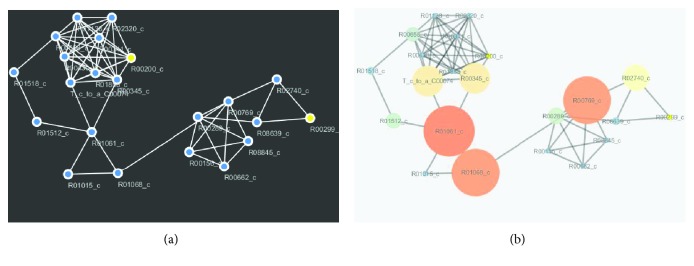
(a) Predicted glycolysis pathway. (b) Predicted essential glycolysis pathway.

**Figure 7:**
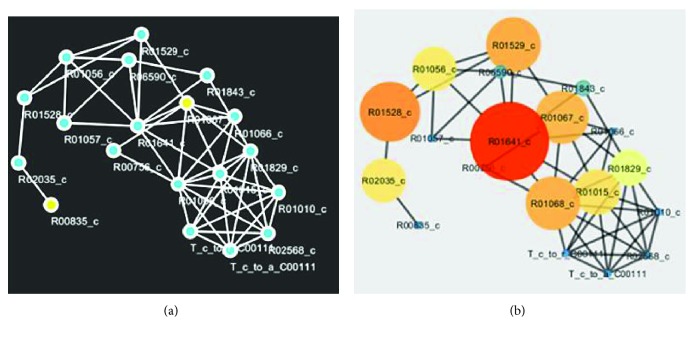
(a) Predicted pentose phosphate pathway. (b) Predicted essential pentose phosphate pathway.

Figures 6(a) and 6(b) are the visualization of the predicted glycolysis pathway where Figure 6(a) represents the unevaluated version of the particular pathway while Figure 6(b) represents the pathways of the betweenness centrality measure. The nodes with the biggest structure in Figure 6(b) represent the predicted essential reactions of the pathway. Similarly, Figures 7(a) and 7(b) are the visualization of the predicted glycolysis pathway where Figure 7(a) represents the unevaluated version of the particular pathway while Figure 7(b) represents the pathways that the betweenness centrality measure has been applied. The biggest nodes in Figure 7(b) denote the predicted essential reactions of the pathway.

## 4. Discussion

Several *P. falciparum* metabolic network reconstructions have been done previously, but this study is aimed at predicting alternative metabolic pathways for energy generation in the malaria parasite via the pentose phosphate and glycolytic reactions using the *k*-shortest path algorithm. The essentiality of these reactions to the survival of these parasites was also evaluated in the predicted models (Table 3) and genes with no human homologs identified (Table 4). Sequence alignment was performed for all the 32 enzymes to identify the existence of homology; two of the enzymes show insignificant similarity with the host. Energy production in apicomplexans involves both the aerobic and anaerobic pathways, and this varies from species to species [32]. *P. falciparum* is a fast growing organism in the human erythrocyte. Therefore, in order to meet its energy need for growth and cell division, it relies on anaerobic oxidation of glucose to generate ATP. This is achieved through a series of enzyme-catalyzed breakdown of glucose to pyruvate, a metabolic process otherwise called the glycolysis or Embden–Meyerhof–Parnas pathway [33]. This process does not require oxygen to generate ATP, but results in the accumulation of pyruvate. While the main purpose of glycolysis is to generate ATP, one of its intermediates, glucose-6-phosphate, is the precursor for the pentose phosphate pathway [34]. This pathway serves an important purpose of generating NADPH and pentose sugars, which are moieties needed for the synthesis of nucleic acids and membrane lipids that are molecular raw materials needed by the parasite for cell division.

Similarly, different stages of malaria parasites have been reported to have different energy requirements while *P. falciparum*-resistant strains have been shown to alter their genetic and metabolic pathways, in order to utilize nutrient requirements for fitness and survival in a drug environment. A recent study identified metabolic changes in mutant parasites, which shows differential transport and utilization of nutrients, as shown in computational prediction that incorporates metabolomics data from sensitive and resistant isolates [35]. Malaria parasite also lacks capability to store carbohydrates [36, 37]; it constantly requires the production of this through glucose and can utilize other carbohydrate precursors scavenged from a host such as UDP-glucose; hence, the identified alternative metabolic pathways that *P. Falciparum* utilizes for survival are crucial for curbing drug resistance and can be targeted as vaccine candidates to reduce malaria transmission. Variable regulation of host UTP-glucose-1-phosphate uridylyltransferase (UDP-glucose phosphorylase), which catalyzes energy-generating reactions such as galactose metabolism and glycogen synthesis, has been reported [34] thereby making it a good target. This was identified as an essential pathway in glycolysis in the present study. Other predictions from this study corroborate the findings from other studies; these are highlighted in Table 3. Amino acid deprivation can alter the growth of *P. falciparum in vitro*; however, it may not completely kill the parasite [38]. This regulation of nutrient requirement to cope with deficit is one of the many survival mechanisms already identified in the parasite. However, other essential amino acid pathways identified can be targeted together to achieve a detrimental effect on the parasite's survival. Our study identified gamma-L-glutamyl-L-cysteine:glycine ligase as an essential reaction for glycine, serine, and threonine metabolism. This finding has been previously reported in other studies [18, 39].

For the cysteine and methionine pathways, hemoglobin digestion is essential for the malaria parasite; L-methionine transport reaction and L-cysteine:2-oxoglutarate aminotransferase were also predicted as essential reactions. The altered transport of nutrients across the membrane may also vary in sensitive and resistant parasites [35]; hence, vaccines or therapies that target these pathways will provide potentially lethal effects on the parasites. Other predicted essential reactions within the glycerophospholipid pathway are 1,2-diacyl-sn-glycerol transport reaction, L-methionine s-adenosyltransferase, and s-adenosyl-L-methionine (only the latter has validated predictions in the literature [40–42]). In the glycolytic pathway, three reactions: D-glyceraldehyde-3-phosphate; D-fructose-1,6-bisphosphate D-glyceraldehyde-3-phosphate-lyase; and UTP:D-fructose-6-phosphate 1-phosphotransferase, were predicted as essential reactions with D-glyceraldehyde-3-phosphate-lyase validated in the literature [43]. Five reactions, namely, inositol-1,4-bisphosphate 1-phosphatase; phosphatidylinositol-3,4-bisphosphate 4-phosphatase; 1-D-myoinositol-3-phosphate phosphohydrolase; CDP-diacylglycerol:myo-inositol 3-phosphatidyltransferase; and inositol-1,3,4-trisphosphate 5/6-kinase, were the predicted essential reactions within the inositol phosphate pathway out of which CDP-diacylglycerol:myo-inositol 3-phosphatidyltransferase is validated [18, 44]. It is noteworthy that D-glyceraldehyde-3-phosphate dehydrogenase has homolog in the human genome (Table 4).

Similarly, our study predicted a metabolite—sedoheptulose-1,7-diphosphate—from the breakdown of xylulose, in the pentose phosphate pathway, which may be utilized by the parasite to scavenge for energy. Only sedoheptulose-7-phosphate:D-glyceraldehyde-3-phosphate glycolaldehyde transferase was predicted as an essential reaction within the pentose phosphate pathway. *P. falciparum* essentially synthesize nucleotides *de novo*. Hence, it cannot salvage pyrimidines from an extracellular environment. This biosynthetic pathway has been identified as a good target for malaria control [45]. For the purine and pyrimidine pathways, five and four reactions were predicted as essential and have been reported in other studies (see details in Table 3). The implications of these hypothesis are that in addition to other predicted pathways, if biologically validated through disruption of genes, encoding the enzymes for these pathways in *P. falciparum* will further elucidate survival and alternate energy generation pathways in the parasite. The hypothesized potential pharmacological enzyme targets essential for the parasite can be targeted to control malaria infection globally.

## 5. Conclusions

In this work, we have been able to predict alternative metabolic paths in the glycolysis and pentose phosphate pathways of *P. falciparum*. We predicted two (2) essential proteins in the glycerophospholipid, purine, pyrimidine, and glycolysis metabolic pathways (Table 4) without homology with a host. With the use of heuristic function to enhance the *k*-shortest path algorithm, we have been able to identify potential biologically relevant paths using a computational graph-based technique which hitherto is less utilized due to its very high false positive result. Biologically targeting these candidate proteins in Plasmodium is recommended to improve understanding of the predicted alternative pathways and the precision of the graph theory-based method.

## Figures and Tables

**Algorithm 1:**
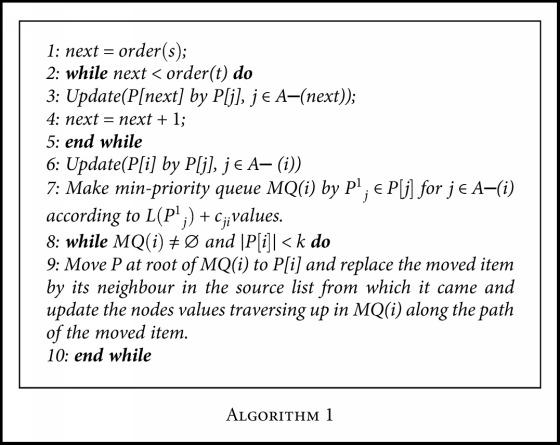


**Algorithm 2:**
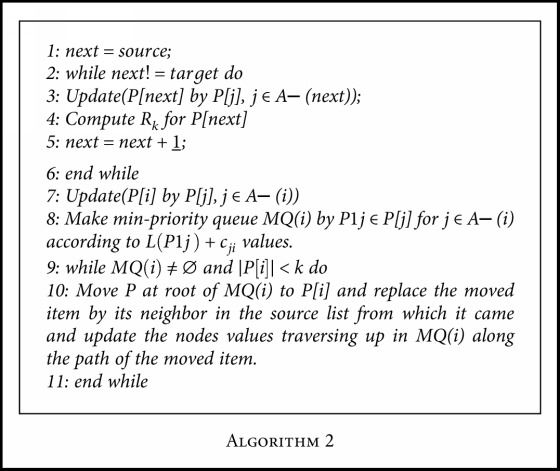


**Table 1:** Time complexity of *T*∗, *K*∗, Yen, Feng, EA, and LVEA algorithms. As shown in the Table 1, the *T*∗ algorithm has better computational performance in terms of running time when compared to others [19].

Algorithm	Time complexity
*T*∗	*O*(*m* + *nk* log *d*)
*K*∗	*O*(*m* + *n* log *n* + *k*)
Lazy variant of Eppstein's algorithm (LVEA)	*O*(*m* + *n* log *n* + *k* log *k*)
Eppstein's algorithm (EA)	*O*(*m* + *n* log *n* + *k* log *k*)
Feng	*O*(*kn*(*m* + *n* log *n*))
Yen	*O*(1/2(*Kn*^3^))

**Table 2:** Computed cost of predicted paths.

*K*	No. of reaction in the path	No. of reaction in annotated pathway	Reward (*R*_*k*_)	Adjusted cost
Glycolysis				
1	9	8	3.5	5.5
2	10	9	4	6
3	11	9	3.5	7.5
4	11	9	3.5	7.5
5	12	10	4	8

Pentose phosphate				
1	8	8	4	4
2	9	9	4.5	4.5
3	10	9	4	6
4	11	11	5.5	5.5
5	11	11	5.5	5.5

**Table 3:** Reactions with the centrality score above the specified level of significance.

Reaction ID	Reaction name	EC number	Biological process	Centrality score	Reference
HBDG_c	Hemoglobin digestion		Cysteine and methionine	0.521053	
T_c_to_a_C00073	L-Methionine transport reaction		Cysteine and methionine	0.442105	
R00896_c	L-Cysteine:2-oxoglutarate aminotransferase	2.6.1.1	Cysteine and methionine	0.426316	
T_c_to_r_C00641	1,2-Diacyl-sn-glycerol transport reaction		Glycerolipid	0.409357	
R02251_r	Acyl-CoA:1,2-diacyl-sn-glycerol O-acyltransferase	2.3.1.20	Glycerolipid pathway	0.324619	
T_c_to_r_C00641	1,2-Diacyl-sn-glycerol transport reaction		Glycerophospholipid pathway	0.42986	
R00177_c	ATP:L-methionine S-adenosyltransferase	2.5.1.6	Glycerophospholipid pathway	0.264179	Oyelade et al. [12]
R02037_R06868 _R06869_c	S-Adenosyl-L-methionine:(methyl)ethanolamine-phosphate N-methyltransferase	2.1.1.103	Glycerophospholipid pathway	0.255603	Choubey et al. [40], Pessi et al. [41], Witola et al. [42]
R00497_c	Gamma-L-glutamyl-L-cysteine:glycine ligase (ADP-forming)	6.3.2.3	Glycine, serine and threonine	0.287619	Chiappino-Pepe et al. [18], Huthmacher et al. [39]
R01061_c	D-Glyceraldehyde-3-phosphate:NAD+ oxidoreductase (phosphorylating)	1.2.1.12 1.2.1.59	Glycolysis pathway	0.552381	
R01068_c	D-Fructose-1,6-bisphosphate D-glyceraldehyde-3-phosphate-lyase (glycerone-phosphate-forming)	4.1.2.13	Glycolysis pathway	0.495238	Wanidworanun et al. [43]
R00769_c	UTP:D-fructose-6-phosphate 1-phosphotransferase	2.7.1.11	Glycolysis pathway	0.490476	
R03427_c	Inositol-1,4-bisphosphate 1-phosphatase	3.1.3.57	Inositol phosphate pathway	0.533333	
R04372_c	Phosphatidylinositol-3,4-bisphosphate 4-phosphatase	3.1.3.66	Inositol phosphate pathway	0.533333	
R01187_c	1D-Myo-inositol 3-phosphate phosphohydrolase	3.1.3.25	Inositol phosphate pathway	0.47619	
R01802_c	CDP-diacylglycerol:myo-inositol 3-phosphatidyltransferase	2.7.8.11	Inositol phosphate pathway	0.419048	Chiappino-Pepe et al. [18], Fatumo et al. [44]
R03429_c	Inositol-1,3,4-trisphosphate 5/6-kinase	2.7.1.159	Inositol phosphate pathway	0.285714	
R01641_c	Sedoheptulose-7-phosphate:D-glyceraldehyde-3-phosphate glycolaldehyde transferase	2.2.1.1	Pentose phosphate pathway	0.328478	
R00720_c	Inosine 5′-triphosphate pyrophosphohydrolase	3.6.1.8 3.6.1.19	Purine metabolism	0.448718	
R01231_c	Xanthosine-5′-phosphate:L-glutamine amido-ligase (AMP-forming)	6.3.5.2	Purine metabolism	0.37833	McConkey [46]
R00576_c	Glutamine-pyruvate transaminase	2.6.1.15	Purine metabolism	0.345098	
R02024_c	2′-Deoxycytidine diphosphate:oxidized-thioredoxin 2′-oxidoreductase	1.17.4.1	Purine metabolism	0.340121	Barker et al. [47], Chakrabarti et al. [48], Lytton et al. [49]
R01135_c	IMP:L-aspartate ligase (GDP-forming)	6.3.4.4	Purine metabolism	0.330982	Eaazhisai et al. [50]
R00328_c	GDP phosphohydrolase	3.6.1.5; 3.6.1.6; 3.6.1.42	Purine metabolism	0.287731	
R00570_c	ATP:CDP phosphotransferase	2.7.4.6	Pyrimidine	0.45977	Oyelade et al. [12], Chiappino-Pepe et al. [18]
R02024_c	2′-Deoxycytidine diphosphate:oxidized-thioredoxin 2′-oxidoreductase	1.17.4.1	Pyrimidine	0.419157	Barker et al. [47], Chakrabarti et al. [48], Chiappino-Pepe et al. [18], Lytton et al. [49]
R00571_c	UTP:ammonia ligase (ADP-forming)	6.3.4.2	Pyrimidine	0.255172	Chiappino-Pepe et al. [18]
R00573_c	UTP:L-glutamine amido-ligase (ADP-forming)	6.3.4.2	Pyrimidine	0.255172	Chiappino-Pepe et al. [18]
R06517_c	Acyl-CoA:sphingosine N-acyltransferase	2.3.1.24	Sphingolipid	0.431917	Gerold and Schwarz [32]
R02251_c	Acyl-CoA:1,2-diacyl-sn-glycerol O-acyltransferase	2.3.1.20	Sphingolipid	0.325708	
R00351_m	Acetyl-CoA:oxaloacetate C-acetyltransferase (thioester-hydrolysis)	2.3.3.1 2.3.3.3	TCA	0.377739	
R02569_m	Acetyl-CoA:enzyme N6-(dihydrolipoyl)lysine S-acetyltransferase	2.3.1.12	TCA	0.281995	Chiappino-Pepe et al. [18]

**Table 4:** Predicted essential genes with no homologs in the human genome.

Gene name	Enzyme name	E.C. number	Biological process
PF3D7_1343000	Phosphoethanolamine N-methyltransferase	2.1.1.103	Glycerophospholipid
PF3D7_1437200	Ribonucleoside-diphosphate reductase	1.17.4.1	Purine/pyrimidine

## Data Availability

The datasets used in this study are available in http://lcsb-databases.epfl.ch/pathways/Gems.

## .

Supplementary Materials Table S1: reactions obtained from the MetaCyc database to fill gaps in the iPfa GEM.
