# Supplementary material for: Computational Identification of Metabolic Pathways of Plasmodium falciparum using the k-Shortest Path Algorithm
Source: Int J Genomics. 2019 Oct 1;2019:1750291. doi: 10.1155/2019/1750291 (PMC6791207; doi:10.1155/2019/1750291)
Supplement: Supplementary Materials — Table S1: reactions obtained from the MetaCyc database to fill gaps in the iPfa GEM. [file 1750291.f1.pdf]

APPENDIX A

Table S1: Reactions obtained from MetaCyc database to fill gaps in the *iPfa* GEM

NAME	**EQUATION**	**EC-NUMBER**	**SUBSYSTEM**
L-threonine aldolase	L-Threonine[c] <=> Acetaldehyde[c] + Glycine[c]	4.1.2.48, 4.1.2.5	Glycine, serine and threonine metabolism
glycine hydroxymethyltransferase	L-Serine[c] + Tetrahydrofolate[c] <=> Glycine[c] + 5,10-Methylene-tetrahydrofolate[c] + H2O[c]	2.1.2.1	Glycine, serine and threonine metabolism
serine-glyoxylate transaminase	Glyoxylate[c] + L-Serine[c] <=>Hydroxypyruvate[c] + Glycine[c]	2.6.1.45	Glycine, serine and threonine metabolism
ornithine cyclodeaminase	L-Ornithine[c] <=> L-Proline[c] + NH3[c]	4.3.1.12	Arginine and proline metabolism
	(S)-1-Pyrroline-5-carboxylate[c] <=> L-Glutamate 5-semialdehyde[c]		Arginine and proline metabolism
aspartate 4-decarboxylase	L-Aspartate[c] <=> L-Alanine[c] + CO2[c]	4.1.1.12	Alanine, aspartate and glutamate metabolism
citrate (Si)-synthase	Acetyl-CoA[c] + H2O[c] + Oxaloacetate[c] <=> Citrate[c] + CoA[c]	2.3.3.1	Alanine, aspartate and glutamate metabolism
glutamine-pyruvate transaminase	L-Glutamine[c] + Pyruvate[c] <=> 2-Oxoglutaramate[c] + L-Alanine[a]	2.6.1.15	Alanine, aspartate and glutamate metabolism
2-oxoglutaramate amidase	2-Oxoglutaramate[c] + H2O[c] <=> 2-Oxoglutarate[c] + NH3[c]	3.5.1.111	Alanine, aspartate and glutamate metabolism
cystathionine gamma-synthase	L-Cysteine[c] + O-succinyl-L-homoserine[c] <=> succinate[c] + L-cystathionine[c] + H+[c]	2.5.1.48	Cysteine and methionine metabolism
cystathionine beta-lyase	L-cystathionine[c] + H2O[c] <=> ammonium[c] + pyruvate[c] + L-homocysteine[c]	4.4.1.8	Cysteine and methionine metabolism
methionine synthase	L-homocysteine[c] + N5-methyltetrahydrofolate[c] <=>	2.1.1.13	Cysteine and methionine metabolism

	L-Methionine[a] + tetrahydrofolate[c]		
5-methyltetrahydropteroyltriglutamate-homocysteine S-methyltransferase	L-homocysteine[c] + N5-methyltetrahydropteroyl tri-L-glutamate[c] <=> L-Methionine[a] + tetrahydropteroyl tri-L-glutamate[c]	2.1.1.14	Cysteine and methionine metabolism
cystathionine gamma-lyase	L-cystathionine[c] + H_2O[c] <=> 2-oxobutanoate[c] + L-Cysteine[c] + ammonium[c]	4.4.1.1	Cysteine and methionine metabolism
inositol-tetrakisphosphate 5-kinase	D-myo-inositol (1,3,4,6)-tetrakisphosphate[c] + ATP[c] <=> D-myo-inositol 1,3,4,5,6-pentakisphosphate[c] + ADP[c] + H+[c]	2.7.1.140	Inositol phosphate (vit B8) metabolism
inositol-1,3,4-trisphosphate 5/6-kinase	D-myo-inositol (1,3,4)-trisphosphate[c] + ATP[c] <=> D-myo-inositol (1,3,4,6)-tetrakisphosphate[c] + ADP[c] + H+[c]	2.7.1.159	Inositol phosphate (vit B8) metabolism
inositol-1,3,4-trisphosphate 5/6-kinase	D-myo-inositol (1,3,4)-trisphosphate[c] + ATP[c] <=> D-myo-inositol (1,3,4,5)-tetrakisphosphate[c] + ADP[c] + H+[c]	2.7.1.159	Inositol phosphate (vit B8) metabolism
inositol-1,4-bisphosphate 1-phosphatase	D-myo-inositol (1,3,4)-trisphosphate[c] + H_2O[c] <=> D-myo-inositol (3,4)-bisphosphate[c] + phosphate[c]	3.1.3.57	Inositol phosphate (vit B8) metabolism
phosphatidylinositol-3,4-bisphosphate 4-phosphatase	D-myo-inositol (3,4)-bisphosphate[c] + H_2O[c] <=> 1D-myo-Inositol 3-phosphate[c] + phosphate[c]	3.1.3.66	Inositol phosphate (vit B8) metabolism
inositol-polyphosphate multikinase	D-myo-inositol (1,4,5)-trisphosphate[c] + ATP[c] <=> D-myo-inositol (1,4,5,6)-tetrakisphosphate[c] + ADP[c] + H+[c]	2.7.1.151	Inositol phosphate (vit B8) metabolism
inositol-polyphosphate multikinase	D-myo-inositol (1,4,5,6)-tetrakisphosphate[c] + ATP[c] <=> D-myo-inositol 1,3,4,5,6-	2.7.1.151	Inositol phosphate (vit B8) metabolism

	pentakisphosphate[c] + ADP[c] + H+[c]		
inositol-trisphosphate 3-kinase	D-myo-inositol (1,4,5)-trisphosphate[c] + ATP[c] <=> D-myo-inositol (1,3,4,5)-tetrakisphosphate[c] + ADP[c] + H+[c]	2.7.1.127	Inositol phosphate (vit B8) metabolism
inositol-pentakisphosphate 2-kinase	D-myo-inositol 1,3,4,5,6-pentakisphosphate[c] + ATP[c] <=>phytate[c] + ADP[c] + H+[c]	2.7.1.158	Inositol phosphate (vit B8) metabolism
